# Proteomic Profiling of the Liver, Hepatic Lymph Nodes, and Spleen of Buffaloes Infected with *Fasciola gigantica*

**DOI:** 10.3390/pathogens9120982

**Published:** 2020-11-24

**Authors:** Rui-Si Hu, Fu-Kai Zhang, Hany M. Elsheikha, Qiao-Ni Ma, Muhammad Ehsan, Quan Zhao, Xing-Quan Zhu

**Affiliations:** 1 College of Animal Science and Technology, Jilin Agricultural University, Changchun 130118, Jilin Province, China; grishu0707@gmail.com; 2 State Key Laboratory of Veterinary Etiological Biology, Key Laboratory of Veterinary Parasitology of Gansu Province, Lanzhou Veterinary Research Institute, Chinese Academy of Agricultural Sciences, Lanzhou 730046, Gansu Province, China; kid372820378@163.com (F.-K.Z.); maxintan1228@gmail.com (Q.-N.M.); mehsan124@gmail.com (M.E.); 3 Faculty of Medicine and Health Sciences, School of Veterinary Medicine and Science, University of Nottingham, Sutton Bonington Campus, Loughborough LE12 5RD, UK; Hany.Elsheikha@nottingham.ac.uk; 4 College of Veterinary Medicine, Shanxi Agricultural University, Taigu, Jinzhong 030800, Shanxi Province, China

**Keywords:** Buffalo, *Fasciola gigantica*, fascioliasis, quantitative proteomics, liver, hepatic lymph node, spleen

## Abstract

In the present study, we used an isobaric tag for relative and absolute quantitation (iTRAQ) proteomics technology to characterize the differentially expressed proteins (DEPs) in the liver, hepatic lymph nodes (hLNs), and spleen of buffaloes infected with *Fasciola gigantica (F. gigantica)*. We also used the parallel reaction monitoring (PRM) method to verify the expression levels of the DEPs in the three infected tissues. At three days post-infection (dpi), 225, 1821, and 364 DEPs were detected in the liver, hLNs, and spleen, respectively. At 42 dpi, 384, 252, and 214 DEPs were detected in the liver, hLNs, and spleen, respectively. At 70 dpi, 125, 829, and 247 DEPs were detected in the liver, hLNs, and spleen, respectively. Downregulation of metabolism was prominent in infected livers at all time points, and upregulation of immune responses was marked in the hLNs during early infection (three dpi); however, no changes in the immune response were detected at the late stages of infection (42 and 70 dpi). Compared to the hLNs, there was no significant upregulation in the levels of immune responses in the infected spleen. All the identified DEPs were used to predict the subcellular localization of the proteins, which were related to extracellular space and membrane and were involved in host immune responses. Further PRM analysis confirmed the expression of 18 proteins. These data provide the first simultaneous proteomic profiles of multiple organs of buffaloes experimentally infected with *F. gigantica*.

## 1. Introduction

The parasitic disease fascioliasis, caused by *Fasciola hepatica* and *F. gigantica*, affects around 17 million people and causes significant economic losses estimated at nearly $3 billion per annum [1,2]. *F. gigantica* has a complex, multi-stage lifecycle, which is similar to *F. hepatica*, but differ only in the type of the intermediate host snail [3]. Buffaloes serve as the definitive host and acquire infection by ingestion of vegetation contaminated with encysted metacercariae. The metacercariae excyst in the intestine into newly excysted juveniles (NEJs), which cross the intestinal wall and migrate across the peritoneum to the hepatic tissue within a few days [2]. The juvenile stages of *F. gigantica* migrate through liver parenchyma for 6–8 weeks and then enter the bile ducts to complete their development into adult flukes [4]. Inside the buffaloes, the flukes feed on hepatic tissue and blood to support their own growth and development. During their migration and development within the host, *F. gigantica* flukes must overcome a variety of biological barriers and produce effector molecules, which alter the gene expression [5,6,7,8] and modulate the immune responses [9,10] of the affected animals.

Control of fascioliasis in buffaloes is important because buffaloes are considered an economically vital livestock species in many developing countries [7,11,12,13,14]. Due to the emergence of drug-resistance [15,16], there is a clear need for the development of vaccines and new flukicide drugs to effectively control fascioliasis. However, developing new therapeutics has been difficult due to the complex lifecycle of the liver flukes and the limited understanding of molecular pathogenesis of the infection compounded with the incomplete knowledge of the precise mode of action or pharmacokinetics of current flukicide drugs [15,16,17].

A previous transcriptomic analysis of buffalo livers revealed that many genes play roles in response to *F. gigantica* infection at different time points after infection [18]. Also, proteomics analysis of buffaloes infected with *F. gigantica* infection also revealed some of the immune mechanisms used by buffaloes to counter the infection [11]. Hepatic lymph nodes (hLNs) and the spleen are two important immune organs, which play key roles in fighting off various infections [19]. The hLNs are located close to the liver in the retroperitoneal space. Also, the spleen serves as the largest lymphoid organ in the body and is anatomically connected to the liver tissue via the portal vein system. How *F. gigantica* infection reshapes protein expression and the cross-talks between these three important body organs during *F. gigantica* infection remains largely unknown.

In the present study, we performed a comprehensive proteomic analysis of the liver, hLNs, and the spleen of buffaloes experimentally infected with *F. gigantica* using an isobaric tag for relative and absolute quantitation (iTRAQ)-based quantitative approach. Along with the stage transformation of the intra-buffalo parasite stages involved in early juvenile three days post-infection (dpi), mid-juvenile (42 dpi), and late juvenile (70 dpi), our analysis revealed global proteomic changes and complex host response to *F. gigantica*. Bioinformatic analysis was used to identify the differentially expressed proteins (DEPs), which play various roles in the pathophysiology of *F. gigantica* infection. The DEPs were used to identify biomarkers associated with immune response and drug metabolism.

## 2. Materials and Methods

### 2.1. Animals and Experimental Infection

The metacercariae of *F. gigantica* were used to infect the buffaloes as previously described [18,20]. Eighteen, 8-10 month-old, buffaloes were purchased from a local buffalo farm in Guangxi Zhuang Autonomous Region, China. Animals were randomly divided into six groups (three buffaloes/group). Each group of buffaloes was housed in a clean buffalo shed and fed daily with water and food. The fecal samples from all buffaloes were examined using a fecal sedimentation technique in order to ensure the absence of fluke eggs. Also, serum samples were tested using an Enzyme-Linked Immunosorbent Assay (ELISA) in order to detect anti-*F. gigantica* IgG and IgM antibodies. All buffaloes were treated with triclabendazole (1 mL, 5% per kilogram body weight) to ensure the absence of any existing or prior infection.

Animals were acclimatized for four weeks, and then three infected groups were inoculated orally with 500 viable metacercariae contained within capsules containing 0.85% sodium chloride (NaCl) solution. Buffaloes in the three uninfected (control) groups were mock-inoculated with 0.85% NaCl solution within capsules but without metacercariae. At three, 42, and 70 dpi, buffaloes were sacrificed, tissue samples including liver, hLNs, and spleen were collected from each animal and washed a few times using phosphate buffer saline. All samples were immediately frozen in cryogenic tubes and stored at –80 °C until use.

### 2.2. Protein Extraction and iTRAQ Analysis

Proteins were extracted from liver, hLNs, and spleen samples at each time point using a lysis buffer (0.1% Sodium deoxycholate, 7 M Urea, 2 M Thiourea, 2mM Ethylenediaminetetraacetic acid, Tris-base (pH = 8), Protease inhibitor cocktail (Sigma-Aldrich, St Louis, MO, USA), 1 mM Phenyl methyl sulfonyl fluoride). Protein concentrations were determined using a Bradford Protein Assay Kit (Thermo Fisher, Waltham, MA, USA), and the extracted proteins were stored at –80 °C. Before iTRAQ labeling, an equal amount of protein from the respective tissue sample from each buffalo was combined into one pooled sample (combining three biological replicates) per group. Approximately 100 μg protein of each pooled sample was digested with Trypsin Gold (Promega, Madison, WI, USA) at 37 °C for ~16 h. Digested peptides were dried using a vacuum centrifuge. The peptides were then labeled using the iTRAQ Reagent-8PLEX Multiplex Kit (Sigma-Aldrich, St Louis, MO, USA). The labeled peptides were incubated at room temperature for 2 h. Differentially labeled peptides were mixed equally and then desalted into polymer-based tubes (Phenomenex, Torrens, CA, USA). The iTRAQ-labeled peptide mix was fractionated in a Waters BEH C18 (4.6 mm × 250 mm, 5 μm) on a L-3000 high performance liquid chromatography (HPLC) system (Rigol, Beijing, China). The tryptic peptides were monitored at UV 214 nm, the temperature of the column oven was set as 37 °C, and the eluent was collected every minute. The eluted peptides of the same replicate were divided into 12 fractions and dried using a vacuum.

Shotgun proteomics of each fraction was performed by nano liquid chromatography tandem mass spectrometry (LC-MS/MS) analysis using an EaSY-nLCTM 1200 ultra HPLC system (Thermo Fisher, Waltham, MA, USA) coupled to an Orbitrap Q extractive HF-X mass spectrometer (Thermo Fisher, Waltham, MA, USA), operating in the data-dependent acquisition mode. Protein identification was achieved using commercial Proteome Discoverer v.2.1 search engine (Thermo Fisher, Waltham, MA, USA) mapped against the closely related *Bos taurus* proteome (UniprotKB release 2017_07). To maximize the accuracy of the analysis, the peptide spectrum matches with >95% confidence, and at least one unique peptide was used for protein identification. Protein abundance ratio with *p*-value < 0.05 and fold change >1.2 or <0.83 were considered as the cut-off values for differentially expressed proteins between infected and control groups. The protein extraction, iTRAQ labeling, protein identification, and quantification were performed at Tianjin Novogene Co., Ltd. (Tianjing, China) (https://novogene.com/). The raw data are available at iprox database (https://www.iprox.org/) under the accession number IPX0002287000.

### 2.3. Bioinformatic Analysis

The g:Profiler Sever (https://biit.cs.ut.ee/gprofiler/) was used for gene ontology (GO) and kyoto encyclopedia of genes and genomes (KEGG) database pathway enrichment analyses. The g:SCS (set counts and sizes) correction method in g:Profilers sever was used to evaluate the level of significance in both GO and KEGG analyses, with the adjusted threshold of FDR (false discovery rate) *p*-value < 0.05. GO enrichment analysis was categorized into biological process (BP), molecular function (MF), and cellular component (CC). Only *p*-value < 0.05 and a protein number ≥10 in each GO term and pathway were considered for further analysis. Significantly enriched GO terms were used to identify the intersecting GO terms between different tissues and between different time points after infection. A minimum of three GO terms from each comparison were selected, and the interacting GO terms were visualized using the pyecharts library (https://pyecharts.org/). For all significantly enriched pathways, ggplot2 package in R (https://www.r-project.org/) was used to show the enrichment results. The subcellular localization of all identified DEPs was inferred using the bologna unified subcellular component annotator (BUSCA) database [21].

### 2.4. Verification of Proteomic Results Using Parallel Reaction Monitoring (PRM)

iTRAQ-based proteomic results were validated by using a sensitive and rapid parallel reaction monitoring (PRM)-based LC-MS/MS approach [22]. A total of 18 randomly selected proteins were analyzed in the liver (three dpi), hLNs (42 dpi), and spleen (three dpi). The protein preparation of the liver, hLNs, and spleen tissue samples was performed separately as described above and used for PRM analysis. Targeted MS peptides were subjected to liquid chromatography electrospray ionization tandem mass spectrometry analysis on a TripleTOF 5600 System (SCIEX, Concord, ON, Canada) following the manufacturer’s protocol, and the PRM raw data were collected. ProteinPilot v.4.2 was used to identify proteins and peptide precursor ions, and the resulting data were visualized using the software Skyline v.4.2. The PRM experiments, including data processing, were performed by Wuhan GeneCreate Biological Engineering Co., Ltd. (Wuhan China) (www.genecreate.com).

## 3. Results

### 3.1. Detection of F. gigantica in Infected Buffaloes

The presence of *F. gigantica* in the liver tissue and bile duct at three, 42, and 70 dpi has been confirmed in infected buffaloes in our previous transcriptome study of *F. gigantica* [20]. In the uninfected buffaloes, no flukes were detected in the liver tissue or the bile duct. Likewise, fluke eggs were not detected in the fecal samples. The serum of uninfected buffaloes was negative for anti-*F. gigantica* IgG and IgM antibodies.

### 3.2. DEPs affected by F. gigantica Infection

**Figure 1:**
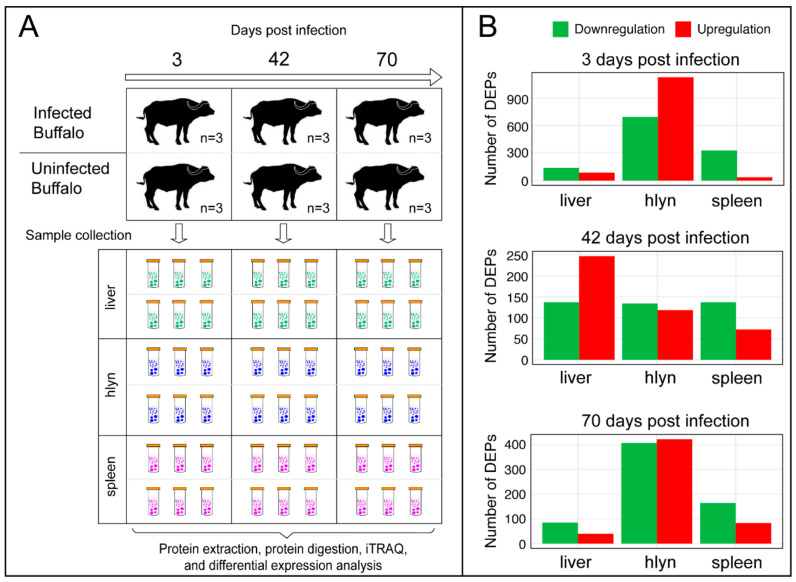
Results of the isobaric tag for relative and absolute quantitation (iTRAQ)-based proteomic analysis of buffalo tissues during the course of *F. gigantica* infection. (**A**) Experimental design showing three infected and three control buffalo groups (three buffaloes/group). Tissue samples from the liver, hepatic lymph nodes (hLNs), and spleen were collected at three, 42, and 70 days post-infection (dpi). Protein of each data (time point and tissue type) was extracted and digested and subjected to iTRAQ analysis and differential expression analysis. (**B**) Proteomic results showing the number of differentially expressed proteins (DEPs) in the liver, hLNs, and spleen at each time point post-infection. Red and green colors represent proteins with increased and decreased expression, respectively.

**Figure 2:**
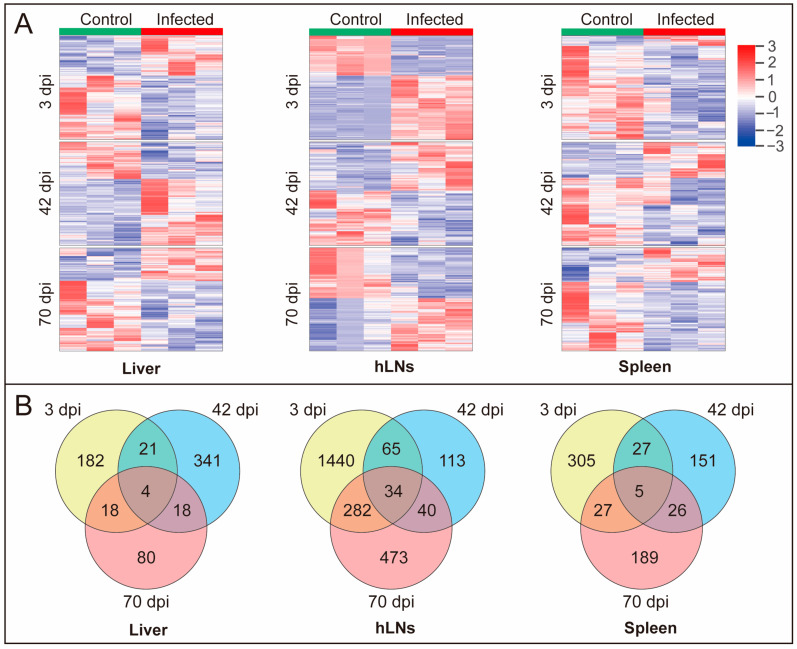
Temporal changes in the buffalo proteins during *F. gigantica* infection. (**A**) Clustering heat maps of the proteins from liver, hLNs, and spleen. In each tissue, heat maps show the expression pattern at three time points (three, 42, and 70 dpi). Columns represent the sample from control and infected groups (three biological replicates per group) and rows represent the DEPs. (**B**) Venn diagrams showing the unique and common DEPs detected in the liver, hLNs, and spleen at three, 42, and 70 dpi.

We used iTRAQ-based proteomics to identify the global alterations of proteins in the liver, hLNs, and spleen in the buffaloes at three, 42, and 70 dpi (Figure 1A). Our analysis revealed 3267, 3672, and 4628 proteins in the liver, hLNs, and spleen, respectively. More than 280,000 spectra were obtained in each infected group. The summary of protein identification data is provided in Table S1. The majority of proteins (80%) from infected and uninfected (control) organs showed coefficient variations <0.4 (Figure S1), indicating the reliability of the proteomic results. The number of the DEPs detected in infected tissues at three, 42, and 70 dpi are shown (Figure 1B). At three dpi, 86, 1127, and 36 DEPs were upregulated, but 139, 694, and 328 proteins were downregulated in liver, hLNs, and spleen, respectively. At 42 dpi, 247, 118, and 72 were upregulated, but 137, 134, and 142 were downregulated in liver, hLNs, and spleen, respectively. At 70 dpi, 40, 422, 83 were upregulated, but 85, 407, and 164 were downregulated in liver, hLNs, and spleen, respectively. However, a total of 4,801 proteins in infected liver, hLNs, and spleen were not differentially altered compared to the uninfected (control) tissues (Table S2). Clustering analysis showed marked differential expression in the proteins of liver, hLNs, and spleen between infected and uninfected buffalo groups at each time point after infection (Figure 2A). Venn diagrams showed that the majority of DEPs were specific for each time point, or there was no overlap between the liver, hLNs, and spleen tissues (Figure 2B). This result reflects the differential response of these tissues to *F. gigantica* during the course of infection. We also performed targeted mass spectrometry analysis using PRM to validate the proteome results, including six randomly selected proteins from the liver (three dpi), six proteins from the hLNs (42 dpi), and six proteins from the spleen (three dpi), respectively. The PRM expression data were consistent with those obtained by iTRAQ data (Figure S2), demonstrating the validity of the obtained iTRAQ proteomic data.

### 3.3. Functional Enrichment Analysis of the Identified DEPs

We performed GO classification to identify the biological functions of the upregulated and downregulated proteins in each of the examined tissue. The identified proteins were classified into the following three categories: BP, CC, and MF. We also performed KEGG pathway analysis. Results obtained from both GO and KEGG analyses are shown (Table S3). The following results of enrichment analysis involve the findings based on the top 20 BP GO terms and pathways.

In the infected liver, upregulated proteins were enriched in the oxidation-reduction process at three dpi, and in the host defense response and tissue repair, such as immune system process, response to wounding, and wound healing at 42 dpi (Table S3). At 70 dpi, upregulated proteins were not significantly enriched in any BP. Interestingly, the infection caused significant downregulation of metabolic processes (Table S3), such as monocarboxylic acid metabolic process, fatty acid metabolic process, and drug metabolism—cytochrome P450.

In the infected hLNs, at three dpi, the significantly altered processes involved the upregulation of immune system process, lymphocyte activation, antigen processing and presentation. We also detected upregulation of immune-related pathways, including leukocyte transendothelial migration (LTM), B cell receptor signaling pathway, and chemokine signaling pathway. Details of the proteins involved in these pathways are listed in Table S4. Interestingly, at 42 and 70 dpi, upregulation of immune-related processes/pathways were not detected. In addition to changes in the host immune response at three dpi, we also detected significant alteration in the metabolism-related processes/pathways, such as downregulation of small molecule metabolic process, oxoacid metabolic process, carboxylic acid metabolic process, and fatty acid metabolism (Table S3). In contrast, we did not observe any downregulated process/pathway at 42 dpi, but there was only one enriched mRNA metabolic process at 70 dpi.

In the infected spleen, the upregulation of protein on three and 42 dpi was not significantly enriched in any BP. However, upregulation and downregulation of proteins simultaneously altered the extracellular space at 70 dpi, but were dominated by the downregulated processes (Table S3). In terms of the pathway enrichment analysis, over the time course of infection, there were no significantly enriched pathways in spleen samples.

**Figure 3:**
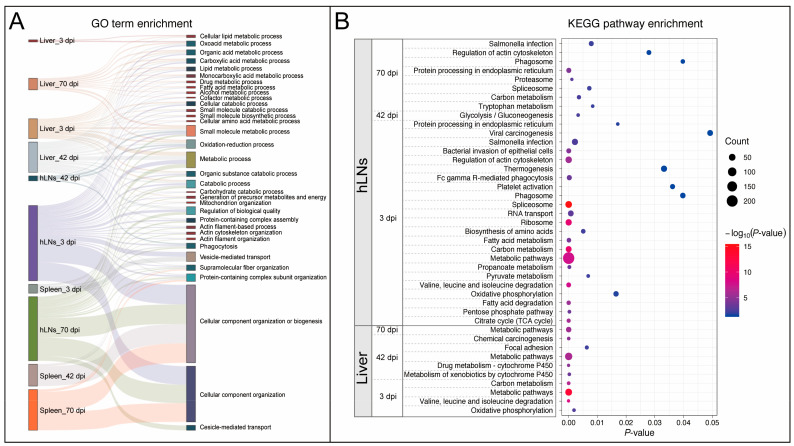
Gene ontology (GO) enrichment and kyoto encyclopedia of genes and genomes (KEGG) database pathway analysis. (**A**) The cross-relationship of GO enrichment results in terms of DEPs in infected liver, hLNs, and spleen at three time points (three, 42, and 70 dpi). (**B**) KEGG pathway analysis showing the enrichment results of DEPs in infected liver and hLNs (*p*-value < 0.05). The DEPs in infected spleen samples were not significantly enriched in any pathway.

We also performed GO and KEGG enrichment analysis of all DEPs (including both downregulated and upregulated proteins). According to the adjusted *p*-value using g:Profiler database, as shown in Figure 3A, the Sankey diagram demonstrated the cross-relationship of the top significantly enriched BP terms. The KEGG pathways of the DEPs at any time point post-infection were not enriched in infected spleen samples (Figure 3B).

### 3.4. Subcellular Localization Analysis of DEPs

**Figure 4:**
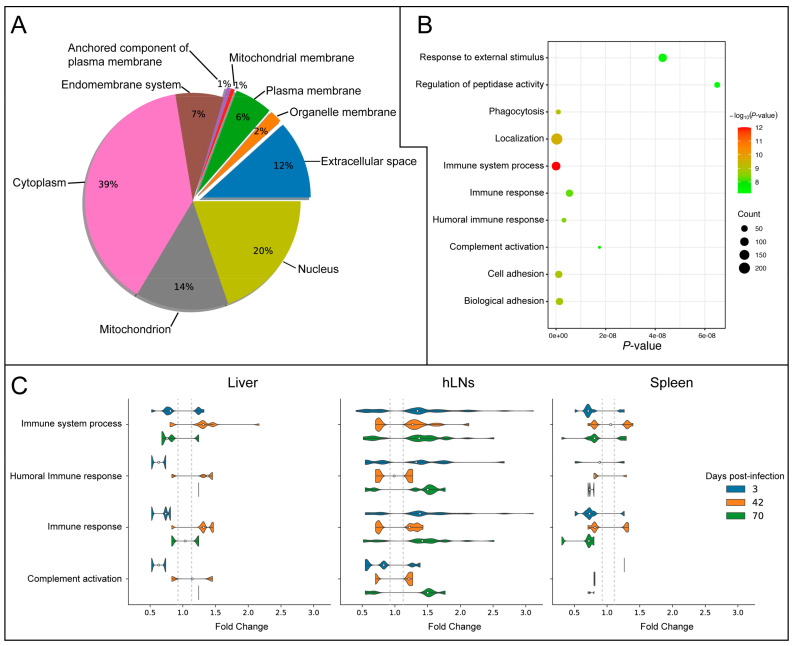
Subcellular localization of the DEPs. (**A**) Pie chart showing the percentage of subcellular locations of the DEPs. (**B**) The top ten biological processes (BPs) of the DEPs related to the membrane and extracellular space. (**C**) The expression patterns of the immune-related DEPs in infected liver, hLNs, and spleen. The *X*-axis label represents the fold change (FC) value of the DEPs in infected versus control groups (FC > 1.2 or < 0.83). The *Y*-axis label denotes four immune-related BPs of interest, representing the three time points.

We used the BUSCA database to predict the subcellular locations of all DEPs. The results predicted that the proteins in all investigated tissues were derived from the cytoplasm (39%), mitochondrion (14%), nucleus (20%), extracellular space (12%), endomembrane system (7%), plasma membrane (6%), organelle membrane (2%), mitochondrial membrane (1%), and the anchored component of the plasma membrane (1%) (Figure 4A and Table S5). Among these, extracellular space and membrane-related proteins represented the potential host molecules that are most likely engaged with the parasite. Therefore, we performed GO enrichment analysis of these extracellular space and membrane-related proteins. The result suggested that these proteins were significantly enriched in host immune-related BPs, such as immune defense, immune system process, humoral immune response, immune response, and complement activation (Figure 4B). Compared to the liver and spleen, protein changes in the hLNs, especially those with increased expression, were more involved in immune-related processes (Figure 4C).

## 4. Discussion

We used iTRAQ-based proteomics approach to investigate the effect of *F. gigantica* infection on the proteomes of the liver, hLNs, and spleen of buffaloes. DEPs were detected in infected tissues at three, 42, and 70 dpi, respectively, providing a global overview of proteins that were sequentially affected during the course of infection (Figure 1B). The differential expression pattern of proteomic profiles identified in the liver in the present study together with transcriptome profiles of liver tissue previously described [18], obtained from the same buffalo samples, suggested that the numbers of dysregulated proteins and transcripts at 42 dpi were higher than those at the other two time points after infection. In regard to the hLNs, we detected a marked increase in the upregulated proteins at three dpi compared to 42 and 70 dpi (Figure 1B). However, spleen samples had higher levels of downregulated proteins at three dpi than the other two time points (42 and 70 dpi) (Figure 1B). These results indicated that these two immune lymphoid organs, hLNs, and spleen, play important roles in the early response to *F. gigantica* infection.

### 4.1. Hepatic Response to F. gigantica Infection

Functional enrichment analyses at three dpi showed that only the oxidation-reduction (redox) process was significantly upregulated in infected livers (Table S3), which might be correlated with electron transmission as a result of redox reaction between buffalo and *F. gigantica* during early infection. At 42 dpi, upregulation of the BPs was involved in host wound healing, response to wounding, and immune system process, demonstrating the liver regenerative capacity to minimize the tissue damage caused by the invasion and migration of juvenile *F. gigantica* flukes. The development and growth of *F. gigantica* within its host involves a series of developmental stages that progress from NEJs to the late juveniles. This stage transformation involves invasive migration through liver parenchyma to the bile duct, which causes significant tissue remodeling. Interestingly, at 70 dpi there were no upregulated BP terms or pathways in the liver. However, the downregulation of metabolism-related processes or pathways was prominent in infected liver across all time points, with some differences between the different times post-infection. For instance, the downregulation of chemical carcinogenesis and drug metabolism-cytochrome P450 was significant at 42 dpi (Figure 3B and Table S3), whereby the enriched functional features were consistent with the previous observation of differentially expressed transcripts in infected liver at 42 dpi [18]. Chemical carcinogenesis is regarded as the primary reagent in the etiology of cancer [23]. Although the link between fascioliasis and liver fibrosis has been documented [24], their association in cancer development remains unclear. In regard to drug metabolism-cytochrome P450, many drug interactions resulted in alteration of cytochrome P450 metabolism pathway [25]; likewise, hepatic enzyme–dimethylaniline monooxygenase (Q8HYJ9) and glutathione transferase enzyme (A1A4L7) were altered in this pathway. The present study showed that their expression levels were downregulated in infected liver at 42 dpi, but not altered at three and 70 dpi. Previously, it has been shown that the anthelmintic triclabendazole increased the expression of Q8HYJ9 (gene symbol: FMO3) [26] and A1A4L7 (gene symbol: GSTM4), enzymes that are involved in drug metabolism [27]. The altered activities of Q8HYJ9 and A1A4L7 during fascioliasis may influence the rate of drug’s uptake or metabolism of the host. However, further confirmation of this observation remains to be confirmed.

### 4.2. More Immune Response in hLNs at Early Compared to Late Stages of Infection

At three dpi the immune-related BP terms or pathways were enriched (Table S3). *F. gigantica* increased the levels of protein expression involved in some pathways, such as the LTM, chemokine signaling pathway, and B cell receptor signaling pathway in infected buffaloes (Table S4). Among these, the activation of the chemokine signaling pathway may be required for the production of relevant cytokines to promote B cell responses and LTM during helminth infection [28,29,30,31,32,33]. In contrast to early infection, changes in the immune responses were not observed in the late juvenile infections (42 and 70 dpi). In other parasites, such as the nematode *Brugia malayi*, parasite-derived molecules could directly inhibit LTM [28], and in the closely related fluke species, *F. hepatica,* the proliferation of leukocytes was decreased in the infected bovine host [29,32]. These results suggested that as infection advances, *F. gigantica* suppressed the immune response as an evasion strategy to promote the establishment of an infection within the buffalo host.

Also, we compared the proteome of hLNs to the previous results of serum proteome in infected buffaloes at the same time points of infection (three, 42, and 70 dpi) [11]. We found that some key proteins related to the complement system and *F. gigantica* infection, including E1BD43 (gene symbol: AOC3; protein: amine oxidase), F1N045 (gene symbol: C7; protein: complement component C7), and F2X047 (gene symbol: LYZ; protein: lysozyme), were differentially expressed in sera of infected buffaloes at all three time points of infection [11]; these proteins were upregulated in infected hLNs at three dpi, but their differential expressions were not observed at 42 and 70 dpi. These findings suggested that the upregulation of those hLNs proteins that, during the early infection stage, might play key roles in the innate immune system and possess antiparasitic activity.

### 4.3. Downregulated Proteomic Signature in Spleen Throughout the Infection Course

A proteomic investigation of *Schistosoma mansoni* infection in an animal model showed that altered spleen proteome modulates host immune responses [34]. In the present study, the altered proteins of the spleen were not significantly enriched in any immune-related response during *F. gigantica* infection. In contrast, we observed that the protein expression in infected spleen was dependent on the time elapsed after *F. gigantica* infection, for example, significant downregulation of vesicle-mediated transport and extracellular space at three dpi, extracellular matrix organization at 42 dpi, and supramolecular fiber organization at 70 dpi (Table S3). It remains to be determined whether and if so to what extent these proteomic changes in the spleen are involved in the buffalo response to *F. gigantica* infection.

### 4.4. Membrane and Extracellular Space Proteins Mediate Host Immune Response

Protein-protein interactions are key to understanding the mechanisms of interaction between *F. gigantica* and the host [7,10,12,14,35,36]. We performed prediction analysis to identify the subcellular locations of the DEPs in the liver, hLNs, and spleen (Figure 4A and Table S5). Bioinformatic analysis indicated that some proteins had functional properties related to the generation of components of membranes and the extracellular space (Figure 4A), suggesting the potential ability of the parasite to interact with these host proteins during infection [37,38,39]. GO enrichment analysis demonstrated that these proteins were enriched in immune-related responses, such as immune system process, humoral immune response, immune response, and complement activation (Figure 4B). This suggests that these BPs are important for host defense mechanisms against *F. gigantica* infection, and their alterations could be induced by the parasite or its effector molecules. Helminths are known to secrete effector molecules like proteins, microRNA cargo, and transfer-RNA fragments [40,41,42]. Hence, elucidation of the role of *F. gigantica*’s secretome in the modulation of the host immune response is warranted.

## 5. Conclusions

The iTRAQ-based proteomic analysis was performed to investigate the protein expression profile in the liver, hLNs, and spleen of buffaloes during *F. gigantica* infection at three, 42, and 70 dpi. This analysis revealed >100 DEPs in each infected tissue; however, the biggest changes were detected in infected hLNs at three dpi (> 1,800 DEPs). Immune-related pathways, such as the LTM, B cell receptor signaling pathway, and chemokine signaling pathway, were activated in infected hLNs at three dpi; however, these were not significantly enriched at the late stages of infection (42 and 70 dpi). We also detected downregulation in the drug metabolism processes, such as drug metabolism-cytochrome P450 in infected liver and the extracellular matrix in infected spleen. Subcellular localization analysis showed that DEPs related to extracellular space and membrane play a role in mediating the host immune response. These data improved our understanding of the molecular events that shape *F. gigantica*’s interaction with multiple immune organs in buffaloes.

## Supplementary Materials

Supplementary Materials can be found at https://www.mdpi.com/2076-0817/9/12/982/s1. Figure S1: The cumulative distribution of CV (Coefficient of Variation) values showing the repeatability of the proteomic data. The *X*-axis represents CV value where CV = (standard deviation/mean) × 100%. The *Y*-axis represents the ratio of the cumulative number of proteins corresponding to the CV value to the total number of proteins. Figure S2: PRM validation of the results obtained by the iTRAQ analysis. The trends of the level of expression of these 18 proteins obtained by iTRAQ and PRM were similar with no significant difference between the results obtained by the two methods. Table S1: Summary of protein identification data in the present study. Table S2: Details of the proteins in infected tissues that were not differentially altered at any time point. Table S3: GO enrichment and KEGG pathway analyses of upregulated and downregulated proteins in each infected tissue. Table S4: Details of the differentially expressed proteins of infected hepatic lymph node involved in immune-related pathways at three days post-infection. Table S5: Subcellular localization of the differentially expressed proteins.
