# Supplementary material for: Proteomic Profiling of the Liver, Hepatic Lymph Nodes, and Spleen of Buffaloes Infected with Fasciola gigantica
Source: Pathogens. 2020 Nov 24;9(12):982. doi: 10.3390/pathogens9120982 (PMC7759843; doi:10.3390/pathogens9120982)
Supplement: Supplementary file 1 [file pathogens-09-00982-s001.zip › 994723-Supplementary Files-final/Figure S2.pdf]

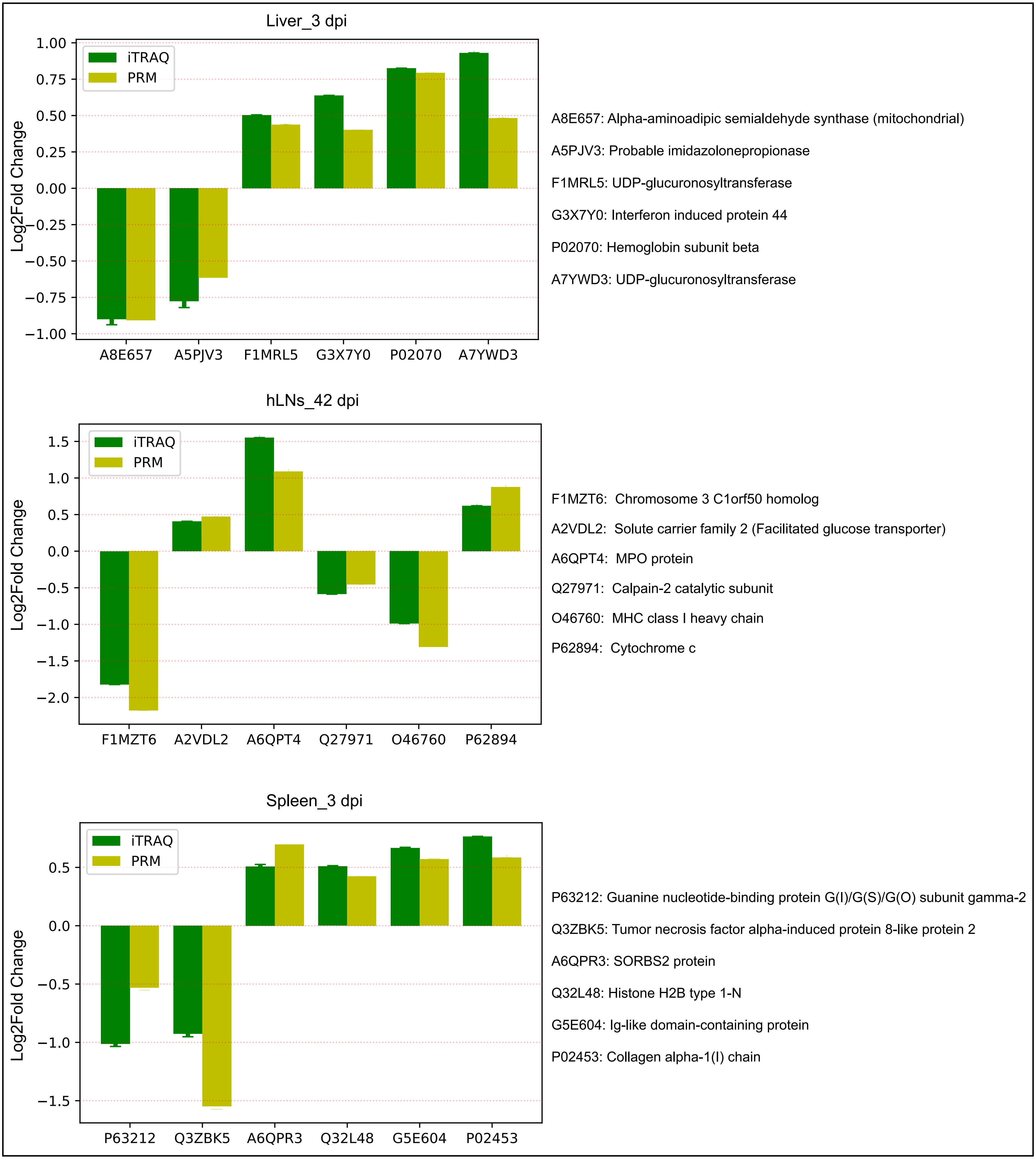

Liver_3 dpi
iTRAQ
PRM
Log2Fold Change
1.00
0.75
0.50
0.25
0.00
−0.25
−0.50
−0.75
−1.00
A8E657
A5PJV3
F1MRL5
G3X7Y0
P02070
A7YWD3
A8E657: Alpha-aminoadipic semialdehyde synthase (mitochondrial)
A5PJV3: Probable imidazolonepropionase
F1MRL5: UDP-glucuronosyltransferase
G3X7Y0: Interferon induced protein 44
P02070: Hemoglobin subunit beta
A7YWD3: UDP-glucuronosyltransferase
hLNs_42 dpi
iTRAQ
PRM
Log2Fold Change
1.5
1.0
0.5
0.0
−0.5
−1.0
−1.5
−2.0
F1MZT6
A2VDL2
A6QPT4
Q27971
O46760
P62894
F1MZT6: Chromosome 3 C1orf50 homolog
A2VDL2: Solute carrier family 2 (Facilitated glucose transporter)
A6QPT4: MPO protein
Q27971: Calpain-2 catalytic subunit
O46760: MHC class I heavy chain
P62894: Cytochrome c
Spleen_3 dpi
iTRAQ
PRM
Log2Fold Change
0.5
0.0
−0.5
−1.0
−1.5
P63212
Q3ZBK5
A6QPR3
Q32L48
G5E604
P02453
P63212: Guanine nucleotide-binding protein G(I)/G(S)/G(O) subunit gamma-2
Q3ZBK5: Tumor necrosis factor alpha-induced protein 8-like protein 2
A6QPR3: SORBS2 protein
Q32L48: Histone H2B type 1-N
G5E604: Ig-like domain-containing protein
P02453: Collagen alpha-1(I) chain
