# Supplementary figures and images for: Proteomic Profiling of the Liver, Hepatic Lymph Nodes, and Spleen of Buffaloes Infected with Fasciola gigantica
Source: Pathogens. 2020 Nov 24;9(12):982. doi: 10.3390/pathogens9120982 (PMC7759843; doi:10.3390/pathogens9120982)

CV Cumulative Curve (Liver)

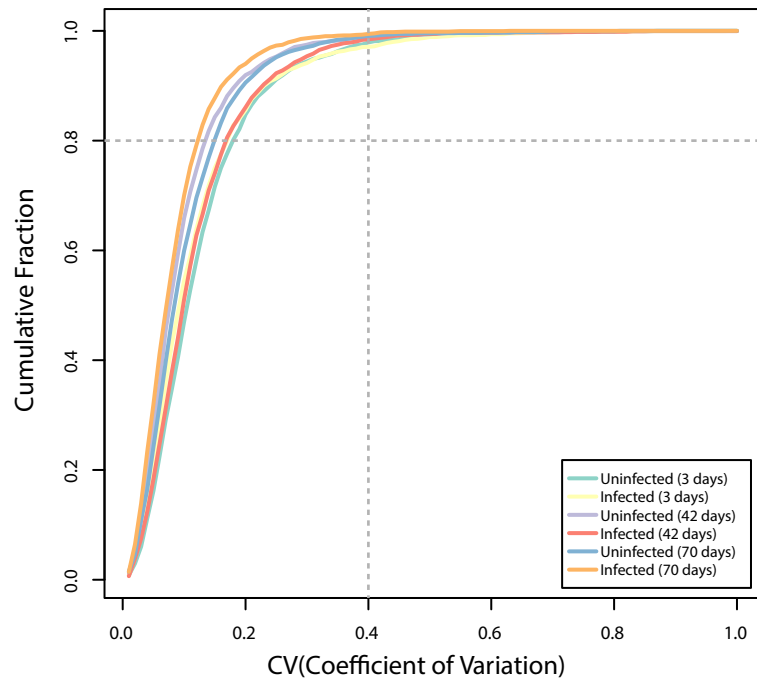

CV Cumulative Curve (hLNs)

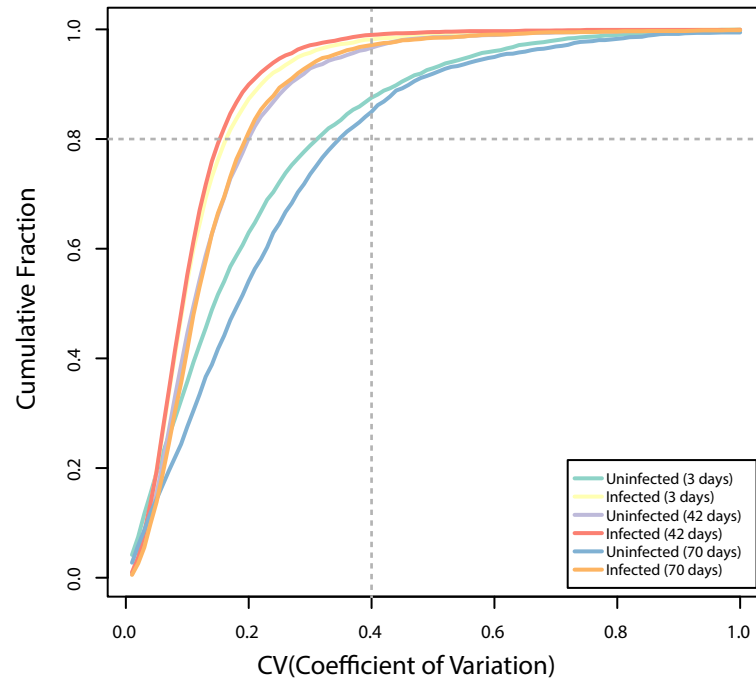

CV Cumulative Curve (Spleen )

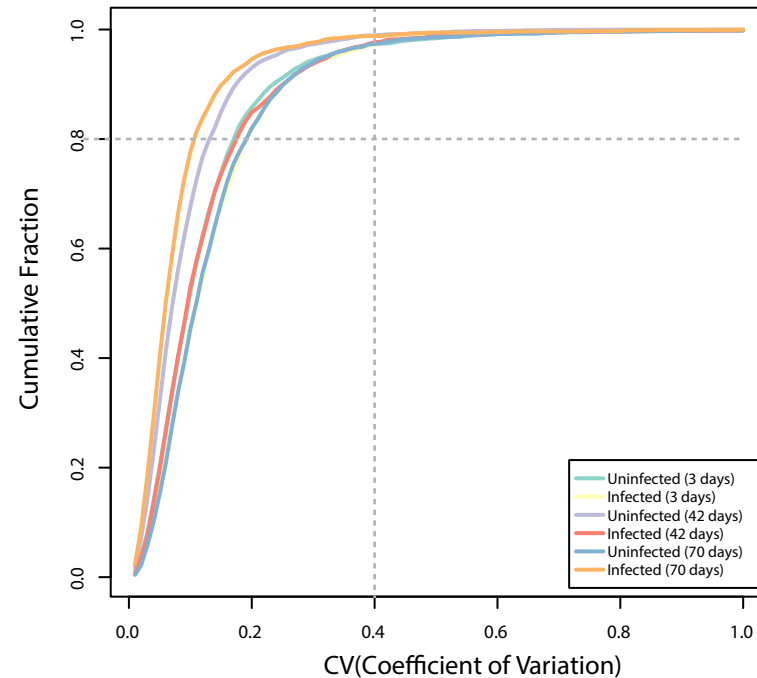

Supplement: Supplementary file 1 [file pathogens-09-00982-s001.zip › 994723-Supplementary Files-final/Figure S1.pdf]
